# Multi‐technical analysis on the antioxidative capacity and total phenol contents of 94 traditional Chinese dietary medicinal herbs

**DOI:** 10.1002/fsn3.689

**Published:** 2018-06-01

**Authors:** Fangting He, Jiayi Chen, Ke Dong, Yun Leng, Jiayi Xu, Pengwei Hu, Yuqin Yao, Jingyuan Xiong, Xiaofang Pei

**Affiliations:** ^1^ Department of Public Health Laboratory Sciences West China School of Public Health and Healthy Food Evaluation Research Center Sichuan University Chengdu China; ^2^ Research Center for Occupational Respiratory Diseases No. 4 West China Teaching Hospital Sichuan University Chengdu China; ^3^ Shenzhen Nanshan Center for Disease Control and Prevention Shenzhen China

**Keywords:** antioxidative capacity, dietary medicinal herbs, total phenol contents, traditional Chinese medicine, Yin‐Yang characteristic

## Abstract

Dietary medicinal herbs (DMHs) are superior reservoirs for natural antioxidants and safe for long‐term consumption. Chinese government recently announced an official list of traditional Chinese DMHs to support civil health promotion. However, antioxidative capacity (AOC) and total phenol contents (TPC) of these listed herbs were not comprehensively and systematically evaluated. In this study, AOC of 94 listed DMHs in water extract were investigated by three techniques including radical scavenging and ferric reducing antioxidative power. The results showed that emblic leafflower, rose, and clove possessed the highest AOC, while TPC was significantly correlated with AOC. While traditional Chinese medicines are categorized by the nature of Yin‐Yang, this study demonstrated that Yin DMHs are statistically correlated with high AOC.

**Practical application:** In this study, AOC and TPC of 94 traditional Chinese DMHs were documented systematically. Emblic leafflower, rose and clove were shown to possess the highest AOC. TPC in traditional Chinese DMHs was significantly correlated with the AOC, while DMHs with Yin characteristic might be used as an indicator to identify potential antioxidative herbs.

## 1 INTRODUCTION

Free radicals create considerable challenges to human health by introducing oxidative stress, which is strongly associated with numerous diseases including cancer, atherosclerosis, neurodegenerative disorders, inflammatory bowel disease, and so on (Saeidnia & Abdollahi, 2013; Shalini, 2015). Free radicals, such as hydroxyl radical and superoxide anion radical, are frequently encountered in the environment and constantly being produced during metabolisms. Antioxidative enzymatic systems including superoxide dismutase (SOD), catalase, peroxiredoxin, thioredoxin, and glutathione systems play vital roles in neutralizing unfavorable free radicals in the body. In addition, antioxidants such as ascorbic acid, tocopherols, tocotrienols, carotenes, and polyphenols are frequently found in vegetables, fruits, eggs, legumes, nuts, and herbs. In food science, accurately measuring and documenting antioxidative capacity (AOC) of various foods is of tremendous importance.

Recently, much attention has been paid to the relationship between antioxidants intake and human diseases (Baures, 2013). Epidemiological studies showed inverse correlations between fruits and vegetables consumptions and the risk of human diseases such as cancer, cardiovascular disease, and diabetes (Scalbert, Manach, Morand, Rémésy, & Jiménez, 2005). Fruits, vegetables, and herbal products are rich in phenolic compounds, known for scavenging free radicals, as well as reducing and chelating pro‐oxidative metal ions. As certain herbal products tend to manifest stronger nutritional and medical properties than fruits and vegetables, World Health Organization (WHO) has recommended dietary medicinal herbs (DMHs) as a complementary therapy for chronic diseases and associated complications (WHO, 2003). Therefore, increasing efforts are being paid to the evaluation of antioxidants derived from natural herbal products.

Herbal products are prominent reservoirs for antioxidants and are extensively applied throughout the world. The intake of medicinal herbal products as food supplements has long been practiced in China based on the empirical notion that “certain food can serve as a mild medication for certain disorder.” Accordingly, plenty of Chinese herbs have been recruited as highly nutrient foods to maintain health, adjust metabolic disturbance, as well as treat diseases in China. In 2002, the Ministry of Health of China officially named 86 traditional Chinese medicines (TCMs) as DMHs to promote general public health (Ministry of Health of China, 2002). In 2014, additional 15 herbs were added to the list of traditional Chinese DMHs. As DMHs possess beneficial properties of both food and medicine, they maintain plenty of advantageous bioactivities and are safe for long‐term consumption at the same time. In China, the concept of “edible medicine” was developed based on the experiences of ancient medical practitioners, neither the Ministry of Health of China nor the academia has announced comprehensive and systematic evaluations on the functions of the listed official traditional Chinese DMHs. Thus, thorough understanding of DMHs is in demand and will benefit the manufacturing of functional foods and dietary supplements.

Several studies have reported that Chinese herbs contain various antioxidants including phenolic acids, flavonoids, and tannins (Cai, Luo, Sun, & Corke, 2004). Although the screening of antioxidative Chinese herbs was performed and the AOC was described, these isolated studies were mainly focused on herbal medicines without clear inclusion criteria for selected plants, leaving out quite a number of officially recognized traditional Chinese DMHs (Jiang et al., 2011; Li et al., 2013; Liu, Qiu, Ding, & Yao, 2008). A few reports demonstrated that ethanol extracts of certain DMHs, such as clove and olive, showed high AOC (Guo, Cheng, Chan, & Yu, 2008; Jiang et al., 2011; Liu et al., 2008). However, these studies employed single analytical method that might be limited and biased, and the AOC of DMHs in water extracts, a more applicable and frequently used sample preparation technique, has not been fully explored.

Therefore, three different techniques, including ferric reducing antioxidative power (FRAP) and radical scavenging assays, were performed in this study to evaluate the AOC of 94 officially listed traditional Chinese DMHs in water extracts. Previous studies suggested that phenolic compounds and the Yin‐Yang nature of TCMs were associated with AOC of medicinal herbs (Dudonne, Vitrac, Coutiere, Woillez, & Merillon, 2009; Liu et al., 2008; Wong, Li, Cheng, & Chen, 2006). Accordingly, the total phenol contents (TPC) of the listed 94 traditional Chinese DMHs were determined by Folin‐Ciocalteu method, and the correlations between TPC and AOC were investigated. In addition, the relationship between AOC and the Yin‐Yang characteristics of traditional Chinese DMHs were also studied. The results of this study may provide comprehensive and systematic documentation of the AOC for the water extracts of 94 traditional Chinese DMHs.

## 2 MATERIALS AND METHODS

### 2.1 Chemicals and materials

Folin‐Ciocalteu phenol reagent, 2,2‐Diphenyl‐1‐picrylhydrazyl (DPPH) radical, chlorogenic acid, 6‐hydroxy‐2,5,7,8‐tetramethylchromane‐2‐carboxylic acid (Trolox), and 2,4,6‐Tris‐(2‐pyridyl)‐s‐triazine (TPTZ) were purchased from Sigma‐Aldrich (St. Louis, MO, USA). All other chemicals and reagent used were of analytical grade. Commercial kits for total antioxidative capacity (ABTs methods) were obtained from Beyotime Biotechnology (Shanghai, China). Traditional Chinese DMHs were purchased from certificated drug stores, including Beijing Tong‐Ren‐Tang (Chengdu, China) and Sichuan De‐Ren‐Tang (Chengdu, China).

### 2.2 Preparation of samples and extracts

All the samples were ground into fine powder and kept cool and dry for analysis. A known value of 0.5 g of the fine powder was extracted with 10 ml of deionized water at 80°C for 30 min in a shaking water bath. The procedure was then repeated one more time. The extracts were cooled down to room temperature, centrifuged at 2,744 g for 10 min and filtered by Millipore filter with a 0.45 μm membrane. The filtrate was stored at 4°C for no more than 24 hr before the determination of AOC and TPC.

### 2.3 Determination of TPC

Total phenol contents was determined by Folin‐Ciocalteu method with chlorogenic acid as the standard (Singleton & Rossi, 1965). Five hundred microliters of the extract was mixed with 2.5 ml of Folin‐Ciocalteu reagent and incubated at room temperature for 3 min. Two milliliters of saturated Na_2_CO_3_ solution (20%, w/v) was added to the mixture, followed by incubation at room temperature for 90 min in the dark. The absorbance of the mixture was measured at 747 nm, and TPC was expressed as mg chlorogenic acid equivalent (mg CAE)/g of dried material weight.

### 2.4 ABTS radical scavenging assay

ABTS assay was conducted under the instruction of total AOC assay kit with ABTS method. Potassium persulfate and ABTS (1:1, v/v) were mixed to generate ABTS stock solution, followed by incubation at room temperature in the dark for 12–16 hr. The stock solution was diluted with phosphate buffer to generate ABTS working solution and the absorbance at 734 nm was adjusted to 0.70 ± 0.05 before the test. ABTS working solution (200 μl) was added to various extracts (10 μl), and the absorbance was measured at 734 nm after incubating for 4 min at room temperature. Phosphate buffer was used as control, and AOC of extracts was expressed by trolox equivalents antioxidant capacity (TEAC) as mM trolox equivalents/g dried material weight.

### 2.5 DPPH radical scavenging activity

Determination of DPPH radical scavenging activity was conducted as previously described (Brand‐Williams, Cuvelier, & Berset, 1995). A known value of 0.1 ml of various extracts was added to 2 ml of DPPH ethanol solution (1.56 × 10^−4 ^M). The mixture was incubated in the dark for 30 min. The absorbance of the solutions was measured at 517 nm, and trolox was used as the standard. DPPH radical scavenging activity was expressed as μmol of trolox equivalents (TE)/g of dried material weight.

### 2.6 Determination of FRAP

FRAP was determined according to the procedure described by Benzie and Strain with slight modifications (Benziea & Strain, 1996). In brief, acetate buffer (0.3 M, pH 3.6), TPTZ (10 mM dissolved in 40 mM of HCL), and ferric chloride (20 mM) were mixed (10:1:1, v/v/v) at 37°C to form FRAP working solution. FRAP working solution (180 μl) was added to various extracts (5 μl). The absorbance of mixed solution was recorded at 593 nm after 3–5 min at 37°C. FRAP value was expressed as μmol Fe (II)/g of dried material weight.

### 2.7 Statistic analysis

All the experiments were conducted in triplicate. Statistical analysis was carried out using SPSS (version 22.0). Results were expressed as means ± standard deviations (*SD*) or medians according to data distributions. The correlations between the AOC and TPC were analyzed by simple linear regression. Differences between AOC and their traditional Chinese Yin‐Yang characteristics were analyzed by rank‐sum test. Significant difference was considered at *p* value <.05.

## 3 RESULTS

### 3.1 AOC of traditional Chinese DMHs

The AOC of traditional Chinese DMHs was determined by three established techniques including ABTS and DPPH radical scavenging assays as well as FRAP assay (Table 1). For ABTS radical scavenging assay, the TEAC values of the 94 listed DMHs exhibited a large variance for up to 2,000 fold, ranging from below detecting limit to 3,619.04 μmol Trolox/g. Emblic leafflower was found to show the highest AOC (3,619.04 μmol Trolox/g), followed by rose (2,716.30 μmol Trolox/g), clove (2,103.29 μmol Trolox/g), and parched flos sophorae (730.76 μmol Trolox/g). For DPPH radical scavenging assay, water extracts of emblic leafflower, rose, and clove showed high AOC of 2,788.22, 1,919.35, and 1,081.05 μmol Trolox/g, respectively, followed by raspberry (584.14 μmol Trolox/g), lotus leaf (389.46 μmol Trolox/g), parched flos sophorae (357.78 μmol Trolox/g), Chinese olive (320.94 μmol Trolox/g), and pepper (314.75 μmol Trolox/g). For lily bub, peach seed, and white hyacinth bean, the ability to scavenge DPPH radicals was not observed. For FRAP assay, the differences of FRAP value among the 94 listed DMHs were significant, ranging from 1.48 to 3,469.87 μmol Fe (II)/g. The DMHs with high FRAP values were ranked as follows: rose, emblic leafflower, clove, raspberry, saffron, parched flos sophorae, lotus leaf, and Sichuan pepper.

**Table 1:** AOC and TPC for 94 water extracts of traditional Chinese DMHs

No.	Vernacular name	Binomial nomenclature	Active organ	TCM characteristics	TPC (mg CAE/g)	FRAP (μmol Fe (II)/g)	DPPH (μmol Tolox/g)	ABTS (μmol Trolox/g)
1	Emblic leafflower	*Phyllanthus emblica* L.	Fruit	Yin, cool	343.38 ± 6.01	2,788.61 ± 265.28	2,488.22 ± 61.74	3,619.04 ± 65.20
2	Clove	*Eugenia caryophyllata* Thunb	Flower	Yang, warm	246.75 ± 6.28	2,659.53 ± 143.43	1,081.05 ± 13.49	2,103.29 ± 24.99
3	Rose	*Rosa rugosa* Thunb	Flower	Yang, warm	193.09 ± 6.35	3,469.87 ± 236.15	1,919.35 ± 32.43	2,716.30 ± 63.36
4	Raspberry	*Rubus chingii* Hu	Fruit	Yang, warm	135.91 ± 0.87	1,274.05 ± 62.26	584.14 ± 13.66	264.85 ± 9.08
5	Parched flossophorae	*Sophora japonica* L.	Flower	Yin, cold	127.39 ± 1.17	933.72 ± 73.15	357.78 ± 4.77	730.76 ± 12.51
6	Seville orange flower	*Citrus aurantium* L. *var. amara* Engl.	Flower	Neutral, moderate	107.97 ± 0.81	617.24 ± 20.12	118.79 ± 0.79	481.74 ± 7.64
7	Sichuan pepper^a^	*Zanthoxylum bungeanum Maxim*.	Peel	Yang, warm	103.68 ± 0.72	727.00 ± 22.12	314.75 ± 6.61	92.21 ± 4.59
8	Red orange	*Citrus reticulata* Blanco	Peel	Yang, warm	103.08 ± 2.30	203.70 ± 12.48	66.25 ± 0.90	534.14 ± 9.38
9	Lotus leaf	*Nelumbo nucifera* Gaertn.	Leaf	Neutral, moderate	94.03 ± 1.09	759.93 ± 37.59	389.46 ± 9.75	713.02 ± 11.62
10	Pawpaw	*Chaenomeles speciosa* (Sweet) Nakai	Fruit	Yang, warm	80.45 ± 0.59	544.90 ± 22.05	287.22 ± 13.30	637.03 ± 32.92
11	Saffron^a^	*Crocus sativus* L.	Stigma	Neutral, moderate	76.71 ± 1.47	966.58 ± 78.03	19.04 ± 1.10	189.96 ± 2.70
12	Galanga^a^	*Alpinia officinarum* Hance	Rhizome	Yang, hot	66.28 ± 1.42	475.20 ± 85.20	259.87 ± 3.77	20.40 ± 0.90
13	Chinese olive	*Canarium album* Raeusch.	Fruit	Neutral, moderate	64.11 ± 0.90	718.44 ± 74.36	320.94 ± 7.87	614.04 ± 12.51
14	Hawthorn	*Crataegus pinnatifida* Bge.	Fruit	Yang, warm	62.09 ± 0.42	457.16 ± 12.82	205.17 ± 10.60	278.47 ± 3.54
15	Chrysanthemum^a^	*Chrysanthemum morifolium* Ramat.	Flower	Yin, cold	58.48 ± 0.98	461.77 ± 38.08	188.27 ± 5.19	59.83 ± 2.86
16	Pueraria	*Puerarialobata* (Willd.) Ohwi	Root	Yin, cool	53.55 ± 1.68	408.62 ± 100.43	61.05 ± 3.86	683.18 ± 8.29
17	Chinese mosla	*Moslachinensis* Maxim.	Aerial parts	Yang, warm	53.52 ± 0.30	56.67 ± 32.18	157.44 ± 1.05	15.32 ± 2.22
18	Houttuynia	*Houttuyniacordata* Thunb.	Whole plant	Yin, cold	42.39 ± 0.44	316.25 ± 11.56	141.18 ± 1.32	244.74 ± 4.45
19	Fructusamomi	*Amomum villosum* Lour.	Fruit	Yang, warm	42.32 ± 1.07	263.35 ± 25.45	131.92 ± 5.86	209.55 ± 5.46
20	Selfheal	*Prunella vulgaris* L.	Fruit cluster	Yin, cold	41.77 ± 1.01	424.51 ± 104.64	124.49 ± 2.50	223.02 ± 10.36
21	Honeysuckle	*Lonicera japonica* Thunb.	Flower	Yin, cold	41.62 ± 0.49	579.70 ± 51.44	191.16 ± 9.48	239.36 ± 6.46
22	Lychnophora^a^	*Sterculia lychnophora* Hance	Seed	Yin, cold	41.56 ± 0.56	10.44 ± 1.34	77.89 ± 2.00	309.07 ± 8.37
23	Seabuckthorn	*Hippophae rhamnoides* L.	Fruit	Yang, warm	38.48 ± 0.32	255.15 ± 17.45	128.26 ± 1.21	240.00 ± 8.88
24	Mint	*Mentha haplocalyx* Briq.	Aerial parts	Yin, cool	35.89 ± 0.20	283.03 ± 19.13	96.13 ± 2.37	177.23 ± 2.39
25	Paniculata	*Microcos paniculata* L.	Leaf	Yin, cool	35.82 ± 1.50	172.02 ± 12.66	73.37 ± 6.29	175.54 ± 5.85
26	Perilla leaf	*Perilla frutescens* (L.) Britt.	Leaf	Yang, warm	33.85 ± 0.42	307.57 ± 27.77	100.76 ± 2.07	159.07 ± 0.79
27	Gardenia^a^	*Gardenia jasminoides* Ellis	Fruit	Yin, cold	33.67 ± 0.31	225.30 ± 36.92	52.83 ± 0.61	44.85 ± 2.79
28	Momordicae	*Siraitia grosvenorii* (Swingle)	Fruit	Yin, cool	33.51 ± 1.38	105.60 ± 21.01	31.20 ± 0.56	161.90 ± 1.64
29	Dried tangerine peel^a^	*Citrus reticulata* Blanco	Peel	Yang, warm	33.23 ± 0.96	15.27 ± 0.86	23.52 ± 0.25	176.60 ± 6.66
30	Witloof	*Cichorium intybus* L.	Whole plant	Yin, cool	33.03 ± 0.54	160.78 ± 2.93	60.65 ± 1.59	92.09 ± 3.64
31	Cinnamon	*Cinnamomum cassia* Presl	Peel	Yang, hot	32.33 ± 0.54	355.97 ± 11.68	214.99 ± 7.10	237.25 ± 10.44
32	Flos lablab album	*Dolichos lablab* L.	Flower	Neutral, moderate	30.63 ± 0.39	177.13 ± 9.22	42.19 ± 0.53	240.66 ± 4.45
33	Cassia seed^a^	*Cassia obtusifolia* L.	Seed	Yin, cold	30.35 ± 0.46	172.55 ± 17.77	32.08 ± 0.39	546.38 ± 19.47
34	Dandelion^a^	*Taraxacum mongolicum* Hand.‐Mazz.	Whole plant	Yin, cold	29.42 ± 0.49	433.28 ± 16.25	106.45 ± 6.07	4.47 ± 0.43
35	Sealwort	*Polygonatumsibiricum* Red.	Rhizome	Neutral, moderate	27.35 ± 0.41	73.34 ± 6.22	25.68 ± 0.89	91.10 ± 12.73
36	Sword bean	*Canavaliagladiata* (Jacq.)DC.	Seed	Yang, warm	24.84 ± 0.27	103.47 ± 19.74	68.98 ± 2.21	106.90 ± 3.64
37	Purslane	*Portulaca oleracea* L.	Aerial parts	Yin, cold	24.65 ± 0.48	198.04 ± 6.58	83.64 ± 1.77	135.75 ± 3.97
38	Mulberry	*Morus alba* L.	Fruit cluster	Yin, cold	24.32 ± 0.30	159.30 ± 10.18	62.80 ± 1.40	134.18 ± 4.10
39	Star anise	*Illicium verum* Hook. f.	Fruit	Yang, warm	23.32 ± 0.35	123.11 ± 11.40	50.91 ± 2.76	72.34 ± 2.53
40	Yellow mustard	*Brassica juncea* (L.)Czern. etCoss.	Seed	Yang, warm	21.43 ± 0.17	111.70 ± 11.76	33.56 ± 0.34	82.96 ± 2.17
41	Radish seed	*Raphanus sativus* L.	Seed	Neutral, moderate	21.14 ± 0.22	98.13 ± 3.55	30.20 ± 0.47	43.38 ± 2.08
42	Fennel	*Foeniculum vulgare* Mill.	Fruit	Yang, warm	20.84 ± 0.38	133.77 ± 0.98	38.70 ± 2.05	99.39 ± 3.14
43	Mulberry leaf	*Morus alba* L.	Leaf	Yin, cold	19.80 ± 0.39	234.23 ± 15.67	42.69 ± 2.25	86.14 ± 1.86
44	Patchouli^a^	*Pogostemon cablin* (Blanco) Benth.	Aerial parts	Yang, warm	18.86 ± 0.75	14.18 ± 2.09	45.34 ± 0.83	99.27 ± 2.24
45	Lophatherum	*Lophatherum gracile* Brongn.	Leaf	Yin, cold	18.44 ± 1.12	113.29 ± 9.65	23.08 ± 0.73	100.50 ± 1.46
46	Field thistle	*Cirsium setosum* (Willd.) MB.	Aerial parts	Yin, cool	18.36 ± 0.21	199.25 ± 11.25	70.28 ± 0.91	108.98 ± 1.65
47	Spina date seed^a^	*Ziziphus jujuba* Mill.var.spinosa (Bunge)	Seed	Neutral, moderate	16.60 ± 0.26	384.60 ± 63.35	7.98 ± 0.57	105.18 ± 1.34
48	Fermented soybean^a^	*Glycine max* (L.) Merr.	Seed	Yin, cool	15.71 ± 0.33	393.29 ± 19.52	14.26 ± 0.15	32.46 ± 1.80
49	Medlar	*Lycium barbarum* L.	Fruit	Neutral, moderate	15.37 ± 0.28	33.37 ± 2.37	17.40 ± 0.72	85.58 ± 2.66
50	Smoked plum	*Prunus mume* (Sieb.) Sieb. etZucc.	Fruit	Neutral, moderate	15.14 ± 0.34	67.72 ± 4.54	24.28 ± 0.31	24.84 ± 0.81
51	Tsaoko	*Amomum tsao‐ko* Crevost et Lemarie	Fruit	Yang, warm	14.01 ± 0.26	169.79 ± 41.44	60.14 ± 1.29	103.06 ± 11.93
52	Turnjujube	*Hovenia dulcis* Thunb.	Seed	Neutral, moderate	12.83 ± 0.16	47.52 ± 2.11	24.56 ± 0.37	77.93 ± 6.71
53	Ginger	*Zingiber officinale* Rosc.	Rhizome	Yang, warm	12.61 ± 0.12	92.42 ± 13.95	26.67 ± 0.48	183.36 ± 3.78
54	Small red bean	*Vigna umbellata* Ohwi et Ohashi	Seed	Neutral, moderate	12.47 ± 0.86	66.23 ± 2.30	25.20 ± 0.78	58.35 ± 2.82
55	Liquorice	*Glycyrrhiza uralensis* Fisch.	Root	Neutral, moderate	12.18 ± 1.21	37.80 ± 1.18	14.18 ± 0.57	108.77 ± 6.08
56	Black pepper	*Piper nigrum* L.	Fruit	Yang, hot	12.14 ± 0.13	56.80 ± 4.42	18.91 ± 0.30	138.42 ± 2.04
57	Turmeric	*Curcuma longa* L.	Rhizome	Yang, warm	11.34 ± 0.04	47.00 ± 0.87	15.20 ± 0.33	58.33 ± 3.75
58	Chinese torreya^a^	*Torreya grandis* Fort.	Seed	Neutral, moderate	9.58 ± 0.13	26.04 ± 2.59	7.07 ± 0.32	139.39 ± 8.50
59	Citron	*Citrus medica* L.	Fruit	Yang, warm	9.49 ± 0.29	51.02 ± 5.80	10.93 ± 0.77	54.93 ± 4.05
60	Chinese angelica	*Angelica sinensis* (Oliv.) Diels	Root	Yang, warm	9.26 ± 0.23	58.66 ± 8.15	17.20 ± 0.67	26.03 ± 0.34
61	Longan meat	*Dimocarpus longan* Lour.	Seed coat	Yang, warm	8.04 ± 0.07	33.97 ± 4.58	16.44 ± 0.63	50.95 ± 3.22
62	Fingered citron	*Citrus medica* L. var. sarcodactylis Swingle	Fruit	Yang, warm	7.54 ± 0.20	37.28 ± 2.85	9.3 ± 1.96	50.83 ± 1.20
63	Chinese date	*Ziziphu sjujuba* Mill.	Fruit	Yang, warm	7.03 ± 0.05	16.03 ± 0.90	9.06 ± 0.24	10.40 ± 0.15
64	Lalang grass rhizome	*Imperata cylindrica*	Rhizome	Yin, cold	6.60 ± 5.25	125.73 ± 6.46	31.32 ± 0.69	38.09 ± 1.27
65	Reed rhizome	*Phragmites communis* Trin.	Rhizome	Yin, cold	6.53 ± 0.14	64.60 ± 5.59	13.69 ± 0.31	32.54 ± 2.04
66	Lotus Seed	*Nelumbo nucifera* Gaertn.	Seed	Neutral, moderate	6.40 ± 0.12	13.11 ± 0.65	1.70 ± 0.40	24.51 ± 0.28
67	Perilla Seed	*Perilla frutescens* (L.) Britt	Fruit	Yang, warm	6.35 ± 0.29	59.47 ± 1.79	24.62 ± 0.35	492.16 ± 10.31
68	Platycodon	*Platycodon grandiflorum* (Jacq.) A.DC.	Root	Neutral, moderate	6.17 ± 0.14	40.03 ± 3.15	11.72 ± 0.37	198.37 ± 3.68
69	Donkey‐hide gelatin	*Equus asinus* L.	—	Neutral, moderate	5.90 ± 0.08	79.05 ± 8.72	10.55 ± 1.71	92.21 ± 9.75
70	Pine Pollen	*Pinus massoniana* Lamb.	Pollen	Yang, warm	5.79 ± 0.14	23.16 ± 1.56	11.32 ± 0.65	41.59 ± 3.44
71	Dahurian angelica	*Angelica dahurica*	Root	Yang, warm	5.69 ± 0.21	29.02 ± 1.35	15.79 ± 1.18	27.83 ± 1.76
72	Sea tangle	*Laminaria japonica* Aresch.	Leaf	Yin, cold	5.64 ± 0.02	50.71 ± 15.55	12.34 ± 0.40	28.05 ± 0.30
73	Ginkgo	*Ginkgo biloba* L.	Seed	Neutral, moderate	5.51 ± 0.27	15.19 ± 0.98	5.47 ± 0.52	59.60 ± 1.14
74	Nutmeg	*Myristica fragrans* Houtt.	Seed	Yang, warm	5.17 ± 0.12	80.59 ± 3.19	19.01 ± 0.45	61.63 ± 1.33
75	White hyacinth bean	*Dolichos lablab* L.	Seed	Yang, warm	4.89 ± 0.22	5.10 ± 0.38	ND	21.44 ± 0.80
76	Bunge cherry seed	*Prunus japonica* Thunb.	Seed	Neutral, moderate	4.80 ± 0.12	12.02 ± 0.61	4.74 ± 0.38	19.99 ± 0.75
77	Corainder	*Coriandrum sativum* L.	Whole plant	Yang, warm	4.62 ± 0.09	32.50 ± 4.33	10.73 ± 0.71	24.38 ± 2.57
78	Piper longum	*Piper longum* L.	Fruit cluster	Yang, hot	4.54 ± 0.14	43.61 ± 9.59	7.41 ± 0.42	29.81 ± 4.44
79	Fragrant solomonseal	*Polygonatum odoratum* (Mill.) Druce	Root	Yin, cold	3.92 ± 0.08	12.27 ± 0.52	5.64 ± 0.81	39.30 ± 19.83
80	Sharpleaf galangal fruit^a^	*Alpiniaoxy phylla* Miq.	Fruit	Yang, warm	3.85 ± 0.01	30.35 ± 22.02	9.91 ± 0.24	136.34 ± 2.66
81	Black sesame	*Sesamum indicum* L.	Seed	Neutral, moderate	3.75 ± 0.04	16.83 ± 0.38	3.79 ± 0.29	16.59 ± 0.20
82	Bitter apricot kernel	*Amygdalus communis* Vas	Seed	Yang, warm	3.04 ± 0.10	8.51 ± 0.64	0.91 ± 0.29	18.69 ± 0.21
83	Malt	*Hordeum vulgare* L.	Fruit	Neutral, moderate	2.91 ± 0.06	14.62 ± 0.51	6.16 ± 0.96	42.66 ± 3.41
84	Ginseng	*Panax ginseng* C. A. Mey.	Rhizome	Yang, warm	2.86 ± 0.14	14.65 ± 3.75	2.56 ± 1.24	27.39 ± 2.66
85	Rhizomakaempferiae^a^	*Kaempferia galanga* L.	Root	Yang, warm	2.71 ± 0.03	21.29 ± 5.88	6.83 ± 0.89	204.74 ± 8.72
86	Peach seed	*Prunus persica* (L.) Batsch	Seed	Neutral, moderate	2.57 ± 0.02	7.11 ± 2.49	ND	16.33 ± 0.51
87	Lily bulb	*Lilium brownii* F. E. Brown var. viridulum Baker	Leaf	Yin, cold	2.31 ± 0.26	9.29 ± 0.70	ND	17.08 ± 1.09
88	Hemp seed	*Cannabis sativa* L.	Fruit	Neutral, moderate	2.25 ± 0.07	13.50 ± 2.74	2.75 ± 0.35	14.59 ± 0.37
89	Longstamen Onion	*Allium macrostemon* Bge.	Stem	Yang, warm	2.25 ± 0.08	8.78 ± 0.69	3.38 ± 0.28	12.30 ± 0.31
90	Yam	*Dioscorea opposita* Thunb.	Rhizome	Neutral, moderate	1.25 ± 0.12	11.20 ± 1.08	7.02 ± 0.36	9.53 ± 1.37
91	Coix Seed	*Coix lacryma‐jobi* L. var. mayuen (Roman.)Stapf	Seed	Yin, cool	0.96 ± 0.01	4.08 ± 0.7523	1.13 ± 0.50	18.47 ± 0.23
92	Tuckahoe	*Poria cocos* (Schw.) Wolf	Sclerotium	Neutral, moderate	0.60 ± 0.01	2.10 ± 0.73	1.45 ± 0.31	1.99 ± 0.32
93	Gordon fruit	*Euryale ferox* Salisb.	Seed	Neutral, moderate	0.48 ± 0.02	10.90 ± 1.09	3.01 ± 0.54	3.03 ± 1.08
94	Honey	*Apis cerana* Fabricius	—	Neutral, moderate	0.27 ± 0.05	1.46 ± 0.19	3.45 ± 0.71	ND

*Notes*

DMHs were sorted based on the TPC values from high to low.

ND: not detectable.

a The ranking of these DMHs varied distinctly by different analytical approaches.

### 3.2 TPC of traditional Chinese DMHs

Total phenol contents was determined according to Folin‐Ciocalteu assay using chlorogenic acid as the standard (Wong et al., 2006). Absorbance at 747 nm was recorded, and TPC was expressed as mg chlorogenic acid equivalent (mg CAE)/g dried material weight (DW) (Table 1). Among the 94 listed traditional Chinese DMHs, the variation of TPC was substantial, ranging from 0.27 to 343.38 mg CAE/g. Emblic leafflower, rose, and clove were found to possess the highest concentrations of total phenols. Some other DMHs such as raspberry, parched flos sophorae, seville orange flower, and Sichuan pepper also exhibited significant amount of TPC (>100 mg/g).

### 3.3 Correlations between AOC and TPC

**Figure 1:**
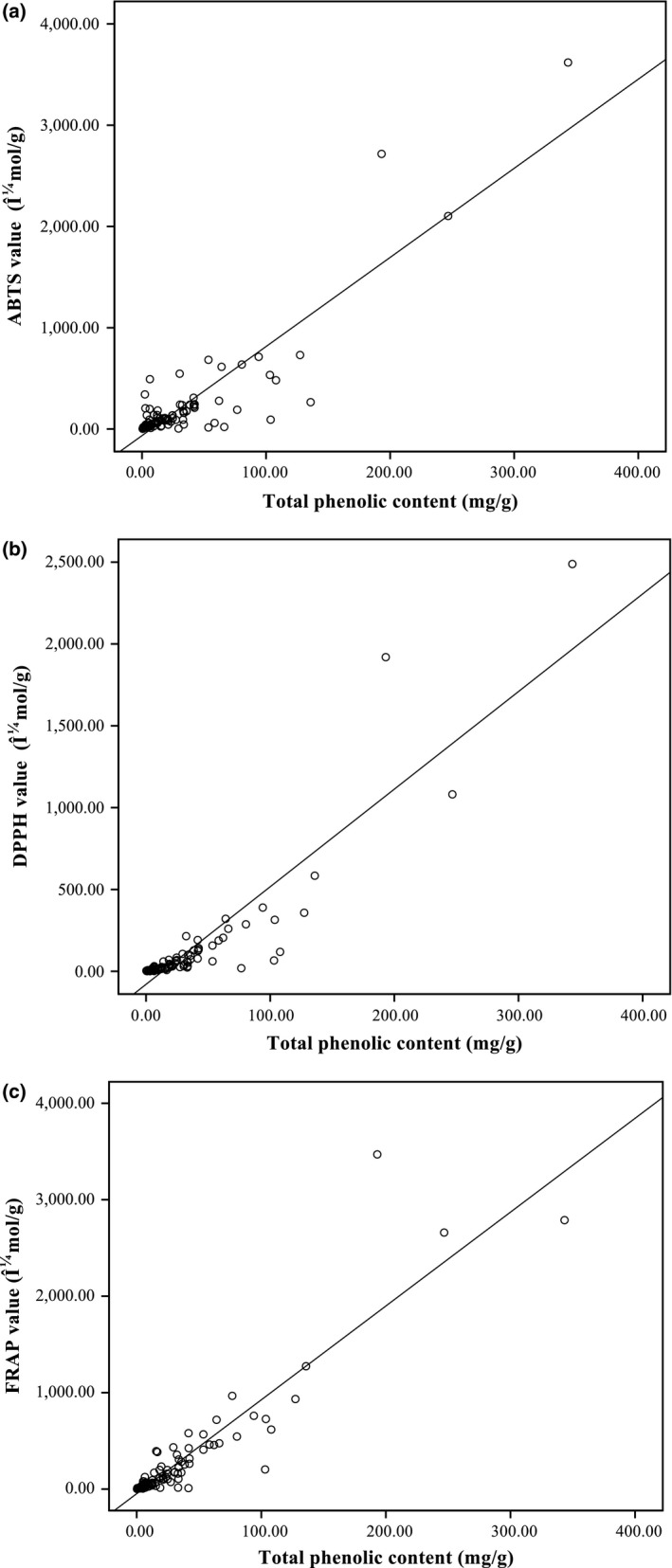
Correlation between the AOC and TPC in 94 water extracts. AOC was measured by the ABTS (a), DPPH (b), and FRAP (c) assays, respectively. AOS: antioxidative capacity; CAE: chlorogenic acid equivalents; TPC: total phenol contents

The correlations between AOC by three techniques and TPC of the 94 listed DMHs were analyzed via simple linear regression. TPC displayed good linear correlation with AOC by all three methods (*r* = .889, .913 and .899 for ABTS, DPPH and FRAP, respectively) (Figure 1). In addition, evaluations were also performed on the correlations between the results generated by different assays (data not shown). This study suggested that there was a strong correlation between AOC and TPC in DMHs, and TPC might serve as a universal indicator for AOC.

### 3.4 Correlations between AOC and Yin‐Yang characteristics

Based on the theory of TCM, traditional Chinese DMHs are classified into three different groups according to their nature of Yin‐Yang (Yin, Yang, and Neutral group). Furthermore, these groups are sub‐divided into five: cold, cool, hot, warm, and moderate subgroup (Ni, 1995). In this study, the Yin‐Yang characteristics and corresponding AOC of 94 listed DMHs were compared (Table 2). In this study, the number of Yin, Yang, and Neutral traditional Chinese DMHs was 27, 40, and 27, respectively. The median for TPC in DMHs with Yin characteristic (cold and cool) was 30.4%, while the medians for TPC in DMHs with Yang and Neutral characteristics were 13.3% and 9.6%. The TPC for DMHs with Yin characteristics were significantly higher than that for DMHs with Yang and Neutral characteristics (*p* < .05), suggesting that Yin characteristics can serve as a potential indicator for traditional Chinese DMHs with high TPC.

**Table 2:** Correlation between TPC and Yin‐Yang characteristics of DMHs

TCM characteristics			Numbers (%)	P25 (mg CAE/g)	P75 (mg CAE/g)	M (mg CAE/g)
Nature	Cold or Cool	Yin	27 (28.7)	15.7	41.6	30.4^a^
Hot or Warm	Yang	40 (42.6)	5.71	41.4	13.3
Moderate	Neutral	27 (28.7)	2.9	21.1	9.6

*Note*

Data were expressed as medians (interquartile range).

a The TPC values for Yin group are significantly different from neutral group. (*p* < .05) P25 and P75: interquartile range; M: medians; Rank Sum Test.

## 4 DISCUSSION

Determination of the AOC in herbal products depends on the conditions of extraction and the analytical techniques. Several methods have been published to measure AOC, including analysis of reducing power, single electron transfer (ET), hydrogen atom transfer (HAT), metal chelation, and others (Shahidi & Zhong, 2015). A couple of studies recruited one of the abovementioned methods to analyze the AOC of natural products including herbal medicines, edible flowers, and spices and showed that clove, cinnamon, pepper, and olive had strong AOC (Guo et al., 2008; Jiang et al., 2011; Li et al., 2013; Liu et al., 2008). However, commonly practiced water extraction was not thoroughly investigated in these studies. In addition, single measurement by one technique is far from sufficient to accurately determine AOC, and a combination of various methods should be considered (Dudonne et al., 2009).

As radical scavenging and reducing power assays are known for their wide application, easy implementation, and repeatable results, the present study employed multi‐technical analysis including ABTS and DPPH radical scavenging assays and FRAP reducing power assay to document the AOC of 94 traditional Chinese DMHs in water extracts. Based on the results, the AOC of these DMHs varied substantially, with over 2,000‐fold differences. In addition, *Rhizoma kaempferiae*, also known as sand ginger, was shown to possess medium AOC (204.74 μmol Trolox/g in ABTS radical scavenging assay, 6.83 μmol Trolox/g in DPPH radical scavenging assay, and 21.29 μmol Fe (II)/g in FRAP analysis). To our knowledge, this is the first documentation reporting the AOC of *Rhizoma kaempferiae*. Furthermore, emblic leafflower, rose, and clove were identified as the top three candidates with potent AOC in the official list of DMHs recommended by Chinese Ministry of Health. These findings would provide solid support for the valuable future application of these traditional Chinese DMHs.

Emblic leafflower, fruit of *Phyllanthus emblica* L., exhibited the highest AOC and TPC among the 94 traditional Chinese DMHs. Emblic leafflower is an important traditional Tibetan herbal medicine and a well‐known tropical fruit in southern China and Asia. Emblic leafflower is a kind of high nutritional herb with strong pharmacological activities, such as antibacterial, immunomodulatory properties, and the protective effects on multiple systems (Baliga & Dsouza, 2011; Bhattacharya, Chaudhuri, Chattopadhyay, & Bandyopadhyay, 2007; Khan, 2009). Hydrolysable tannins were suggested to be responsible for the AOC of emblic leafflower (Yang & Liu, 2014). Rose, the bud of Rosa rugosa Thunb., had the second strongest AOC in the official list of DMHs. *Rosa rugosa* Thunb. is a member of traditional Uygur medicine and acts as the main ingredient in many prescriptions to treat blood conditions, fatigue, and diabetes (Liu, Tanga, Zhao, Xin, & Aisa, 2017; Seo et al., 2015). Volatile oils, flavonoids, and anthocyanin components are the key ingredients accounting for its bioactivities (Gu et al., 2013). Clove, the bud of *Syzygium aromaticum* (synonym: *Eugenia cariophylata*), also demonstrated relatively high TPC and potent AOC. Clove has been widely used as food preservatives, aromatic condiments, and traditional medicine for centuries (Cortés‐Rojas, de Souza, & Oliveira, 2014). Kumatakenin, a flavonoid recently isolated from cloves, was shown to display anti‐cancer effects by inducing apoptosis of ovarian cancer cells and inhibiting alternative activation of tumor‐associated macrophages (Woo, Ahn, Jang, Lee, & Choi, 2017). Eugenol, α‐humulen, β‐pinene, and limonene were the active components found in clove essential oil (Cortés‐Rojas et al., 2014).

In the present study, the AOC of 94 traditional Chinese DMHs in FRAP, DPPH, and ABTS assays were compared and ranked, respectively. The results showed that DMHs with extremely high AOC (emblic leafflower, rose, clove) ranked similarly regardless of the analytical techniques. However, other DMHs with relative lower AOC ranked differently in ABTS assay compared to FRAP and DPPH assays, suggesting that the ability of DMHs to scavenge various radicals was different. Comparing to a previous related research, over 30 herbal medicines were overlapped, and the present study evaluated the AOC for additional 60 DMHs (Li et al., 2013). AOC by FRAP and DPPH assays in this study were generally higher than those reported by Li et al., and the rankings for several DMHs were different. For instance, parched flos sophorae, pueraria, and perilla leaf demonstrated relative strong antioxidative capacity in this study, while Li et al. showed that these DMHs had low antioxidative capacity. This is possibly due to the differences in the temperature used for water extraction. When comparing to another research on AOC, 56 DMHs were overlapped with the present study (Liu et al., 2008). However, the AOC and TPC for pawpaw and raspberry showed distinct variances between this study and report by Liu et al. A likely explanation is that ethanol extraction was used in the previous study while water extraction was recruited in this study.

Although the active components of emblic leafflower, rose, clove, and many other traditional Chinese DMHs were reported, previous studies also indicated that phenolic compounds could significantly contribute to the AOC of medicinal herbs (Dudonne et al., 2009; Liu et al., 2008; Wong et al., 2006). In this study, significant linear correlations were found between TPC and AOC in ABTS, DPPH, and FRAP assays. Positive correlations may reflect the sensitivity of the analytical methods and indicate possible active components. This result suggested that TPC could be utilized as an indicator for AOC in natural products.

In addition, previous studies suggested that traditional Chinese Yin‐Yang characteristic might be linked to the antioxidant–oxidant balance described in modern medicine (Ou, Huang, Hampsch‐Woodill, & Flanagan, 2003). Herbal products with Yin characteristic were rich in polyphenols with high AOC (Ou et al., 2003). Yin‐Yang is the core theory of TCM, which emphasizes the balance, harmony, and homeostasis of whole body. In the development of TCM, traditional herbs with different characteristics are employed to treat diseases, restore physiologic functions, and maintain Yin‐Yang balance. The TCM medicinal theory describes four natures, five flavors, compatibility of medicines, and toxicity. Four natures refer to the four characteristics of herbs (warm, hot, cold, and cool), reflecting the response tendency of herbs to the entity. In recent decades, researchers have investigated the biological effects of TCM characteristics based on modern biomedical sciences, suggesting that different characteristics may participate in the regulation of central nervous system, immune system, and energy metabolism (Zhang & Wang, 2014). However, significant discrepancies existed among most of the studies and further systematic research is needed.

Studies on the relationship between AOC and the characterization of herbal TCM were relatively rare. In this study, relationship between TCM characteristics of these herbs and their TPC was explored. Traditional Chinese DMHs with Yin characteristics showed significantly higher TPC values than DMHs with Yang or Neutral characteristics. While TPC was significantly correlated with the AOC and DMHs with Yin characteristic were rich in TPC, both TPC and Yin characteristic might be utilized as potential indicators for identifying herbal products with high AOC. Our results were in accordance with Ou and Liao (Ou et al., 2003; Liao, Banbury, & Leach, 2007), but inconsistent with the other (Wong et al., 2006). Different results may be attributed to different inclusion criteria of herbs, edibility of the TCM, and analytical approaches. The consistency between traditional insights of TCM and mechanisms of action poses significant challenges for natural products research. Accordingly, our systematic research utilizing three widely accepted techniques would shed light on the characterization and application of traditional Chinese DMHs, a specific and critical category of TCM.

## 5 CONCLUSION

In summary, the AOC and TPC were measured, documented, and compared for the water extracts of 94 traditional Chinese DMHs granted by the Ministry of Health of China. This study established a comprehensive and systematic multi‐technical evaluation on officially endorsed DMHs, proposed TPC as a universal indicator for estimation of AOC, and associated traditional Chinese Yin‐Yang characteristics with TPC. The main findings of this study were emblic leafflower, rose, and clove possess the most potent AOC; inconsistency in AOC existed when using different approaches; traditional Chinese Yin DMHs with high TPC manifested high AOC. Therefore, this study could provide support for future epidemiological researches and dietary guidelines and may serve as a footing stone for healthy dietary guidelines, support further epidemiological researches, and contribute to the identification of potential antioxidative herbs. Further analysis on the chemical compositions, active components, and biological activities of these traditional Chinese DMHs are needed for better understanding and utilization.

## ETHICAL STATEMENT

The authors have no conflict of interest to declare. This work does not involve any human or animal studies.
